# Contemporary Clinical Outcomes and Healthcare Utilization in Older Adults with Acute Myeloid Leukemia: Insights from a Single-Center Retrospective Cohort

**DOI:** 10.3390/cancers18193230

**Published:** 2026-10-07

**Authors:** Tina Y. Zhang, Alex Y. Ge, Shyam A. Patel, Poorva Bindal, Jan Cerny, Andrew Gillis-Smith, Muthalagu Ramanathan, Sakiko Suzuki, Laurie Pearson

**Affiliations:** 1 Division of Hematology/Oncology, Department of Medicine, UMass Memorial Medical Center, UMass Chan Medical School, Worcester, MA 01655, USA; 2 Department of Medicine, UMass Memorial Medical Center, UMass Chan Medical School, Worcester, MA 01655, USA

**Keywords:** older patients, acute myeloid leukemia, clinical outcomes, healthcare utilization

## Abstract

Background: Acute myeloid leukemia (AML) predominantly affects older adults; however, patients 75 years of age and older are often not candidates for curative-intent treatments. Data on their treatment patterns, survival outcomes, and healthcare utilization are limited, and we evaluated these outcomes among patients 75 years of age and older with AML. Methods: We retrospectively analyzed 97 patients aged 75 years and older managed at our academic safety-net hospital between 2016 and 2025. Results: Median overall survival (mOS) was 95 days for all patients. Receipt of any AML-directed therapy was associated with longer mOS compared with no leukemia-directed treatment (144 days, 95% CI: 98–184 vs. 17 days, 95% CI: 7–50; *p* < 0.001). Among patients with 2024 ELN adverse-risk/*TP53*-mutated disease, mOS was 88 days (95% CI: 34-NE) with AML-directed therapy and 44 days (CI: 36-NE) without treatment (*p* = 0.2). Forty-four percent of patients were enrolled in a hospice at their time of death, and an additional 40% were managed with comfort measures as inpatients. Median duration of hospice or comfort care was 2 days (IQR: 1–5), with no difference based on whether patients received any AML-directed treatment. Fifty-one percent of patients died in the hospital. Conclusions: Our findings highlight real-world challenges for older adults with AML, including the need for more effective treatments for patients with *TP53*-mutated AML. AML-directed treatment was associated with longer survival without a decrease in the time spent in hospice care, and the risk of in-hospital mortality remains elevated for this population.

## Simple Summary

Many older adults with acute myeloid leukemia (AML) cannot receive intensive treatment because of comorbidities or frailty. Newer, lower-intensity treatments have expanded options for these patients. In this retrospective analysis, we examined treatment patterns, survival, and healthcare utilization in patients ≥75 years treated at our institution. We found that adding venetoclax to hypomethylating agents was not associated with longer survival compared with hypomethylating agents alone, potentially reflecting treatment selection bias or a smaller sample size. Receipt of any AML-directed treatment was associated with longer survival compared with supportive care alone, except in patients with adverse risk or *TP53*-mutated disease who were not eligible for intensive chemotherapy. Nearly half of patients died in the hospital, and hospice and comfort-focused care were often initiated only in the final days of life. These findings highlight the need for more effective treatments for adverse-risk patients and earlier integration of palliative and hospice care.

## 1. Introduction

Acute myeloid leukemia (AML) is a disease that disproportionately affects older patients. The median age of diagnosis in the United States is 69 years, and incidence peaks between 80 and 85 years. While survival for younger patients has improved dramatically between the 1980s and 2010s, patients 60 years and older have seen only modest improvements in survival, and 5-year overall survival remains less than 5% among patients 70 years and older [1]. Our prior analysis using the National Cancer Database (NCDB) showed that median overall survival (mOS) only improved marginally between 2004 and 2021 for patients 80 years and older [2].

Older patients with AML are more likely to have high-risk mutations. In a retrospective analysis of patients aged 75 years and older treated with induction chemotherapy in a cooperative group study, only 3 out of 151 patients had favorable-risk cytogenetics [3]. Commonly mutated genes include *TET2*, *DNMT3A*, *SRSF2*, *NPM1*, *ASXL1*, *FLT3*-ITD, *IDH2*, and *TP53* [3,4]. In particular, *TP53*-mutated AML is associated with poor outcomes. To date, clinical trials evaluating venetoclax and other targeted agents in combination with hypomethylating agents (HMAs) have not demonstrated improved outcomes over HMA monotherapy in patients with *TP53*-mutated AML [5,6,7,8,9,10]. Retrospective analyses have similarly not demonstrated improved survival with more intensive treatment approaches in *TP53*-mutated myeloid neoplasms [11,12,13]. We recently reported that, in a single-center retrospective analysis, patients with *TP53*-mutated AML who were not candidates for allogeneic stem cell transplant (ASCT) were not shown to have longer survival with AML-directed therapy compared with best supportive care but experienced significantly greater healthcare utilization [14].

Historically, older patients with AML had limited treatment options, and a substantial proportion of patients did not receive AML-directed therapy. An analysis of the Surveillance, Epidemiology, and End Results (SEER) database from 2000 to 2009 showed that approximately 60% of patients older than 66 years did not receive any AML-directed therapy following diagnosis [15]. Similarly, our prior analysis of the NCDB found that the majority of octogenarians and nonagenarians did not receive treatment for leukemia [2]. Data from a single-institution experience showed that while survival outcomes in patients 70 years and older did not differ significantly between those treated with higher-intensity compared with lower-intensity chemotherapy, patients managed with best supportive care alone had significantly shorter overall survival [16].

Despite novel therapy options offering more favorable risk/benefit profiles, treatment of patients 75 years and older remains challenging due to comorbidities, frailty, and functional impairment, which can limit treatment tolerability and increase the risk of treatment-related complications. These patients are often not candidates for curative-intent treatments such as induction chemotherapy and ASCT. HMAs have been available for patients with myelodysplastic syndrome (MDS) since 2005 and were initially used off-label for patients with AML unfit for intensive chemotherapy [17]. Venetoclax, a BCL-2 inhibitor, was granted accelerated approval by the FDA for treatment of AML in combination with HMAs for patients older than 75 years or unfit for intensive chemotherapy in 2018 [7]. Other targeted options have since emerged, including FLT3 inhibitors, mutant IDH1/2 inhibitors, and most recently, menin inhibitors.

Despite the increased incidence of AML in older patients, research focused on this heterogeneous and high-risk population remains limited. Older adults with AML are frequently underrepresented in clinical trials, and, when included, may represent a fitter population than the real-world older adult patient population due to restrictive trial eligibility criteria [18,19,20]. Although retrospective studies have described treatment patterns and survival outcomes in older adults with AML, additional real-world data are needed to characterize outcomes in the context of the evolving therapeutic landscape, which includes the increasing use of lower-intensity and targeted treatment approaches [21,22,23,24,25]. This study aims to characterize real-world treatment patterns, survival outcomes, and healthcare utilization among patients 75 years and older in the era of expanded, lower-intensity therapeutic options.

## 2. Methods

### 2.1. Study Design

A retrospective, single-institution analysis of patients with AML treated at an academic safety-net hospital was performed. This study was approved by the UMass Chan Institutional Review Board under study protocol (IRB#: H00019393) for retrospective analysis of patients with AML and was performed in accordance with the Declaration of Helsinki.

### 2.2. Patient Selection

The study cohort consisted of patients aged 75 years and older at the time of AML diagnosis between 1 January 2016 and 31 August 2025. AML diagnosis was established according to the World Health Organization (WHO) 2016 revised 4th edition criteria [26]. Patients were identified through a retrospective search of the institutional electronic medical record (EMR) database using ICD-10 codes beginning with C92.x and C95.x. All identified records were manually reviewed to assess eligibility. Patients with myelodysplastic syndrome with excess blasts, mixed phenotype acute leukemia, and acute promyelocytic leukemia as per the WHO 2016 revised 4th edition criteria were excluded [26].

### 2.3. Study Variables

The primary objective of this study was to describe real-world survival outcomes and treatment patterns among older patients with AML treated at our institution. Secondary objectives included the evaluation of healthcare resource utilization based on treatment patterns. Demographic, clinical, treatment, and healthcare utilization data were extracted from the electronic medical records. Patients with shared care at other institutions and patients who were treated prior to comprehensive integration of the electronic medical record in 2016 were excluded from analyses of transfusion data.

Patients with missing data for a given variable were excluded from the analyses involving that variable. ECOG PS and ELN risk stratification were exceptions: ECOG PS was unavailable for 32 patients and was retained as an “unknown” category, while patients for whom 2022 or 2024 ELN risk stratification could not be determined were retained as “unable to assess”. All 97 patients were included in the multivariable Cox regression analysis.

Overall survival was defined as the number of days from AML diagnosis to death by any cause. Event-free survival (EFS) was defined as the time from AML diagnosis to relapse or death from any cause, whichever occurred first. Patients who were alive at the end of the study period were administratively censored at the study end date. Overall survival and EFS were analyzed by age at diagnosis, sex, cytogenetic and molecular risk characteristics, Eastern Cooperative Oncology Group (ECOG) performance status (PS), best response, and year of diagnosis. Patients were classified using both 2022 European LeukemiaNet (ELN) risk stratification based on cytogenetic and molecular profiles as well as 2024 ELN risk stratification for patients unfit for intensive chemotherapy. Among patients not considered candidates for intensive treatment, *TP53* mutation is the sole criterion defining 2024 ELN adverse risk. Therefore, among patients not treated with induction chemotherapy, *TP53*-mutated disease and 2024 ELN adverse-risk disease represent the same subgroup. Patients who were treated with induction chemotherapy were excluded from analyses using 2024 ELN risk stratification. Best response was determined using 2022 ELN response criteria. Composite complete response (CRc) included all patients achieving complete response, complete response with partial hematologic recovery, and complete response with incomplete hematologic recovery (CRi).

The median number of treatment cycles for first-line treatment, modifications to first-treatment, and subsequent lines of therapy were compared by age at diagnosis and ECOG PS. Healthcare utilization outcomes were assessed from diagnosis until death, loss to follow-up, or the end of the study period and included the mean and median number of total packed red blood cell (pRBC) and platelet transfusions following diagnosis as well as transfusions within the first 30 and 60 days following diagnosis. Total inpatient days (including the initial diagnosis admission if applicable), intensive care unit (ICU) days, and palliative days were compared based on whether patients received AML-directed treatment, whether patients underwent ASCT, and first-line treatment. Palliative days were defined as days enrolled in hospice care or managed with comfort measures only (CMO) in the inpatient setting.

### 2.4. Statistical Analysis

All statistical analyses were performed using R (version 4.6.0, R Foundation for Statistical Computing, Vienna, Austria). A *p*-value of <0.05 was considered statistically significant. mOS was estimated using the Kaplan–Meier method, and log-rank *p*-values were calculated. Median follow-up time was estimated using the reverse Kaplan–Meier method. Not applicable (NA) indicates that a statistical comparison was not applicable. Where upper 95% confidence intervals (CIs) could not be estimated because the survival curve did not reach the required threshold during follow-up or because of an insufficient sample size or events, these values are reported as not estimable (NE). A sensitivity analysis was performed for the comparison of mOS in treated versus untreated patients by excluding patients who did not receive AML-directed treatment because of clinical deterioration. Cox proportional hazards models were used to adjust for age, year of diagnosis, the 2024 ELN risk category, first-line treatment, and whether patients were diagnosed as an inpatient in the analysis of mOS. The median number of treatment cycles was compared using Kruskal–Wallis tests. The median number of transfusions, inpatient days, ICU days, and palliative care days were compared using Kruskal–Wallis tests. For comparisons across first-line treatment groups, Kruskal–Wallis tests provided global *p*-values, and pairwise comparisons were not performed following a non-significant global test. Nonparametric tests were used rather than parametric t-tests and ANOVA. Means and standard deviations for healthcare utilization measures are reported in the Supplementary Materials for descriptive purposes.

A sensitivity analysis was performed to account for differences in time at risk when evaluating healthcare utilization. Time at risk was defined as the interval from AML diagnosis to death, loss to follow-up, or the end of the study period, whichever occurred first. Utilization rates were calculated by dividing the total number of transfusions, inpatient days, and ICU days by the patient’s time spent at risk in months. Utilization rates were compared between patients who received AML-directed therapy and patients who did not using Wilcoxon rank-sum tests and across first-line treatment groups using Kruskal–Wallis tests. Pairwise comparisons were not performed when the global Kruskal–Wallis test was not statistically significant. The single patient who received gemtuzumab ozogamicin was excluded from the comparisons of first-line treatment because of the small sample size.

Chi-squared tests were used to compare treatment rates across years, the distribution of first-line treatment across years, the proportion of patients requiring ICU admission, and the proportion of patients who died as an inpatient.

## 3. Results

### 3.1. Clinical Outcomes

Ninety-seven patients were included in the analysis, and the median age at diagnosis was 81 years (range: 75–92). The median follow-up time was 94 days (95% CI: 69–135). No patients were excluded from the survival analysis due to missing data or loss to follow-up. Sixty patients (62%) in the cohort were male, and 37 (38%) were female (Table 1). Forty-eight percent of patients were 75–79 years old at diagnosis, 32% were 80–84 years, and 20% were 85 years and older. Eighty percent of patients received AML-directed treatment. Twenty-four percent of patients were diagnosed with AML in 2016–2018, 23% were diagnosed in 2019–2021, and 54% were diagnosed in 2022–2025 (Table 1). Forty-seven percent of patients had a documented ECOG PS of 0 or 1 (Table 1). Only four patients had an ECOG performance status of three or higher; ECOG PS 3 was associated with a significantly shorter mOS compared with ECOG PS 0–2 in pairwise analyses. ECOG PS data were missing for 32 (33%) patients.

**Figure 1:**
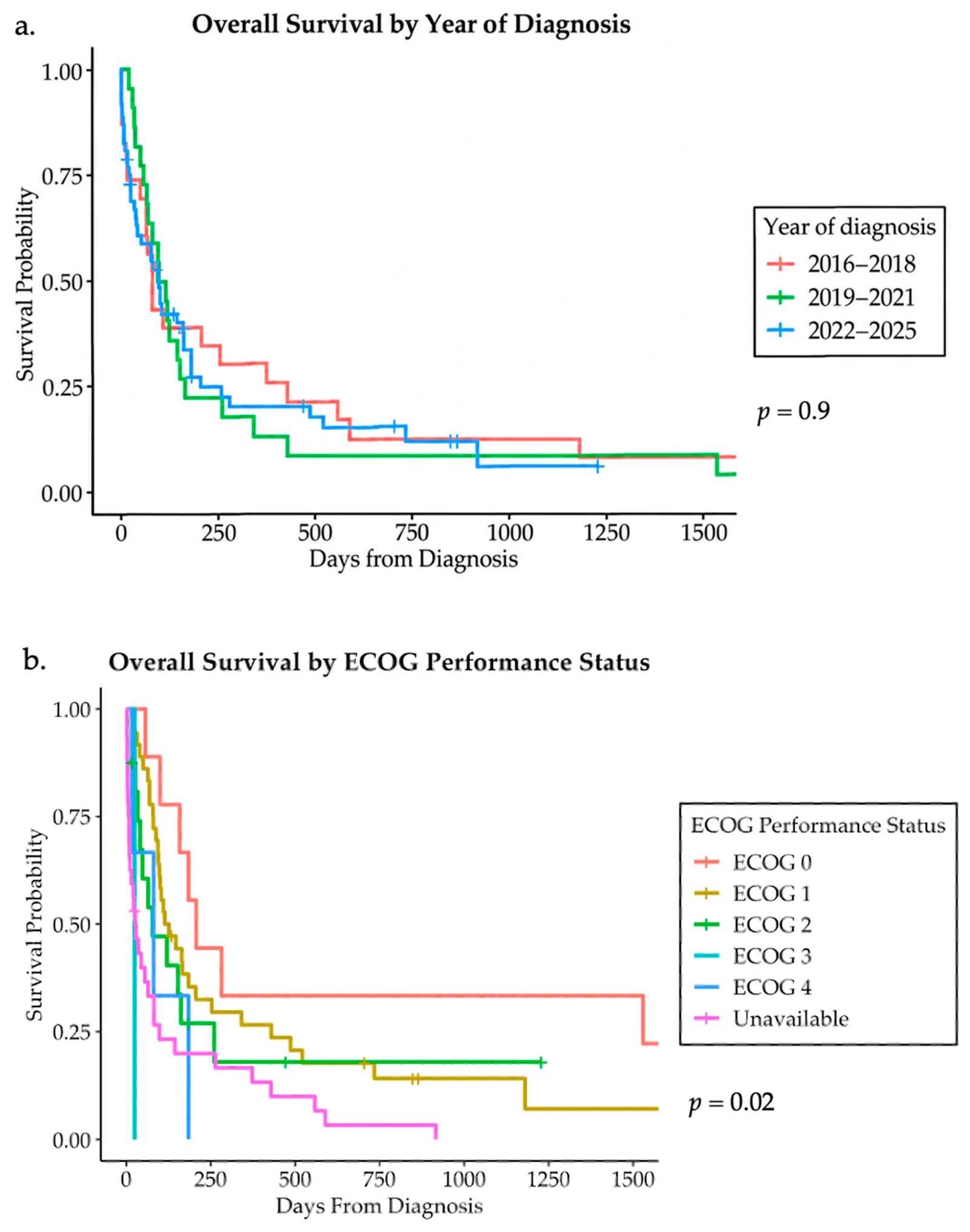

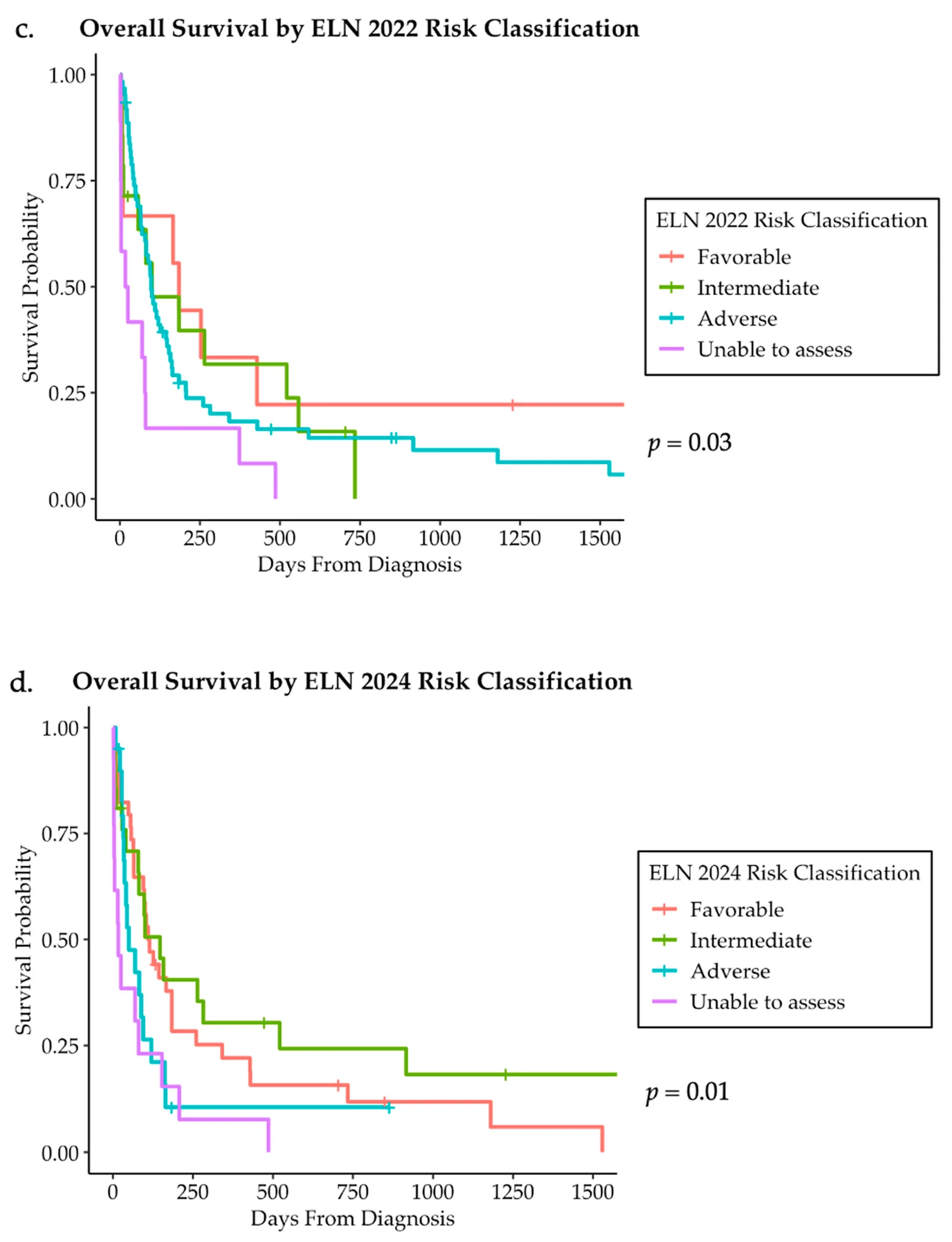

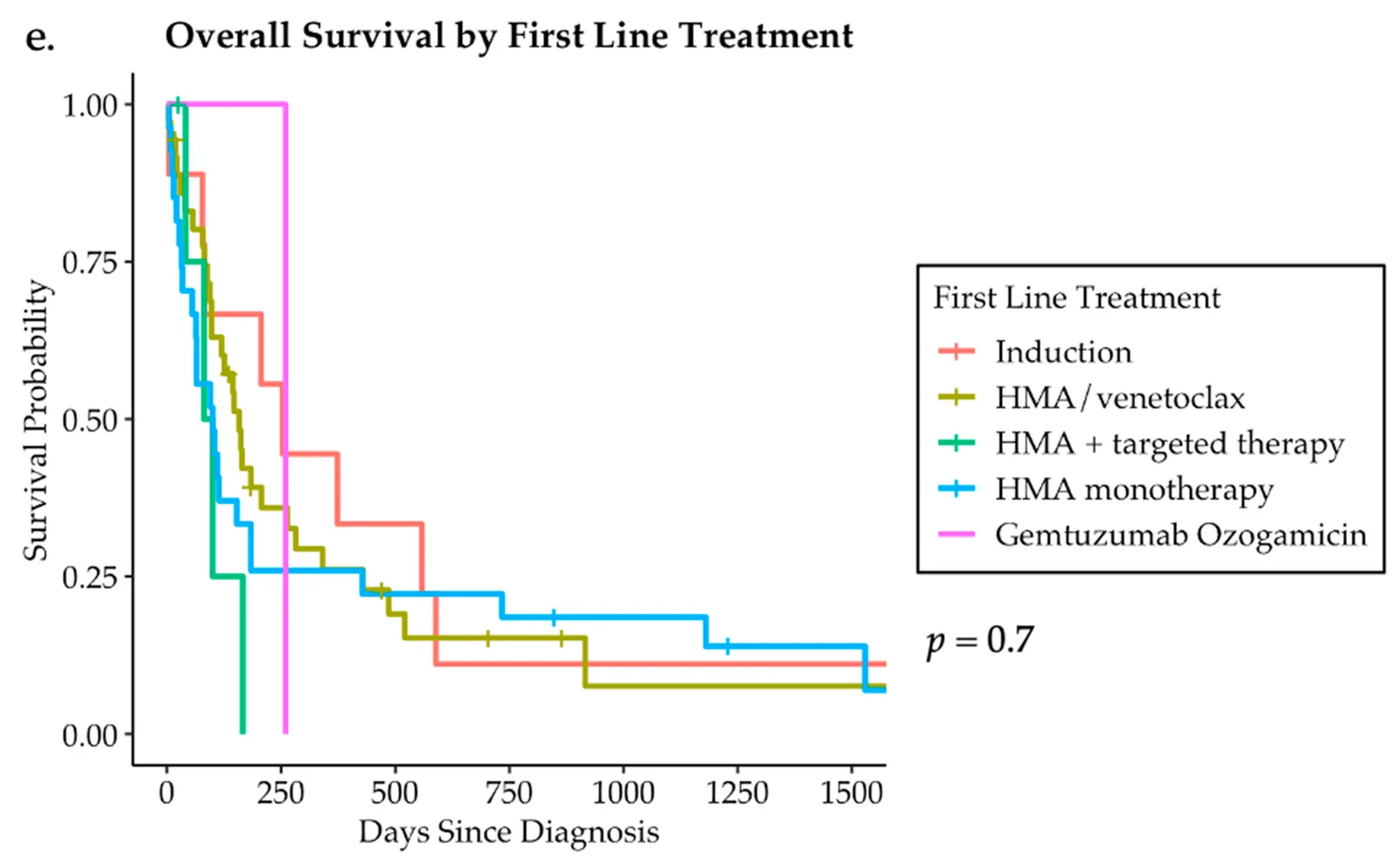
Kaplan–Meier curves for median overall survival (mOS) according to (**a**) year of diagnosis; (**b**) ECOG performance status; (**c**) 2022 ELN risk stratification; (**d**) 2024 ELN risk stratification; and (**e**) first-line treatment. Abbreviations: ECOG = Eastern Cooperative Oncology Group; HMA = hypomethylating agent; ELN = European LeukemiaNet.

**Figure 2:**
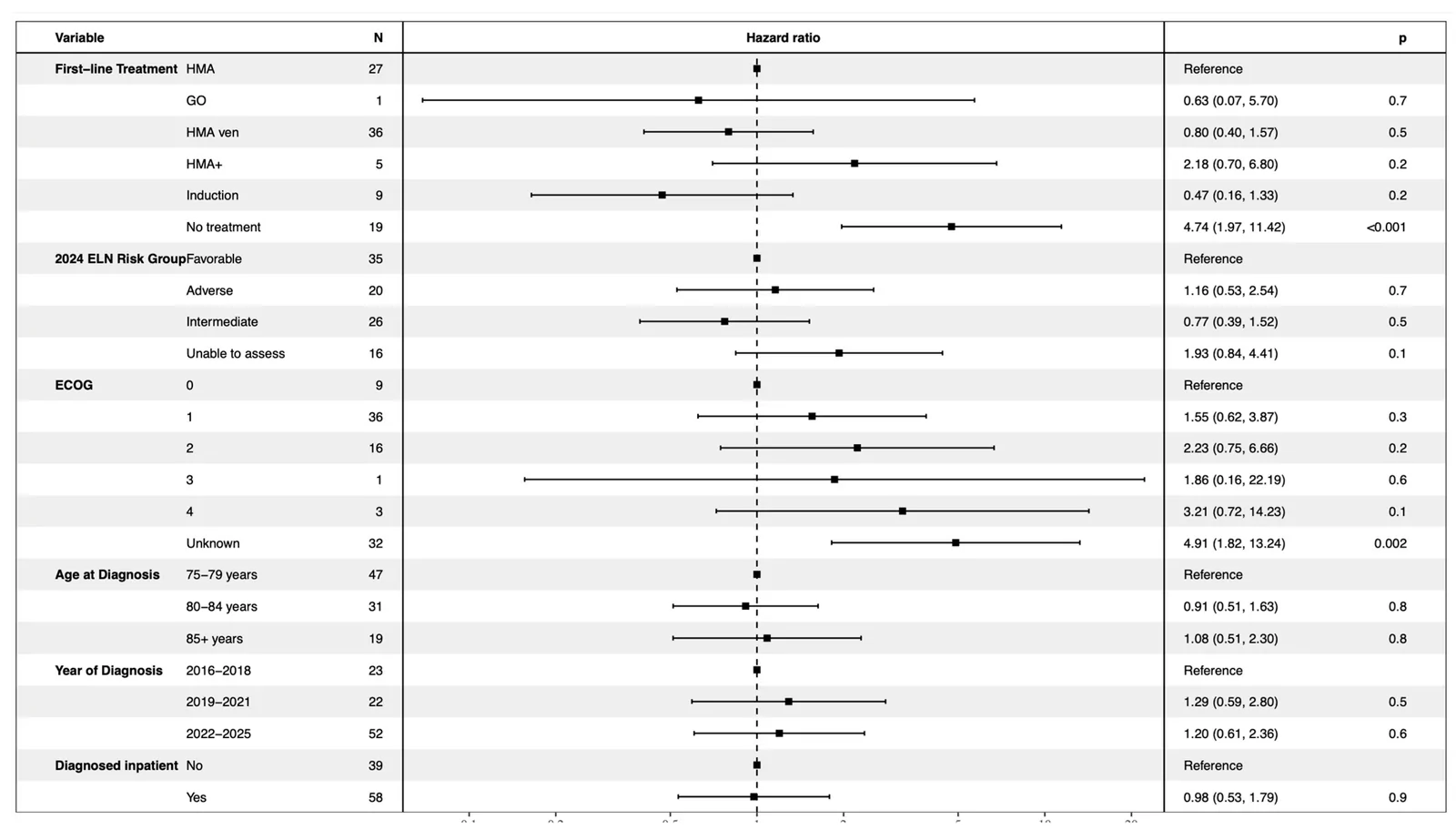
Multivariate Cox regression analysis of mOS by first-line treatment, risk stratification, ECOG performance status, age, diagnosis year, and whether diagnosis occurred as an inpatient. Abbreviations: mOS = median overall survival; ECOG = Eastern Cooperative Oncology Group; ELN = European LeukemiaNet; GO = Gemtuzumab ozogamicin.

Median OS for all patients was 95 days (95% CI: 78–147; Table 1). mOS was not significantly different between male and female patients or among the three age groups, and mOS did not change significantly over time (Table 1 and Figure 1a). Additional EFS analyses are provided in Supplemental Table S1. Nineteen patients did not receive any AML-directed therapy following diagnosis. Six patients who were offered treatment declined. Thirteen patients were not offered treatment. Of these 13 patients, five patients experienced clinical deterioration during inpatient admission at the time of AML diagnosis, six patients were determined to be poor treatment candidates due to competing comorbidities including advanced dementia and metastatic solid tumor malignancy, and two patients had limited treatment options because they developed AML while receiving HMA for MDS and were not candidates for intensive chemotherapy. Both patients had *TP53*-mutated disease. Among all patients, AML-directed therapy was associated with a significantly longer mOS compared with no AML-directed treatment (144 days, 95% CI: 98–184 vs. 17 days, 95% CI: 7–50; *p* < 0.001; Table 1), and this association was maintained when patients who did not receive treatment due to clinical deterioration were excluded from the analysis. Pairwise comparisons showed that treatment with HMA monotherapy (35% of treated patients), HMA and venetoclax (46%), HMA and another targeted agent (6%), or induction chemotherapy (12%) as first-line therapy were all associated with significantly longer mOS compared with no AML-directed treatment (*p* < 0.05, Figure 1e). There were no significant differences in mOS between the first-line treatment groups. These findings remained consistent after controlling for age, 2024 ELN risk stratification, ECOG performance status, year of diagnosis, and whether patients were diagnosed in the inpatient setting (Figure 2).

Patients who were classified as having favorable risk AML by the 2022 ELN criteria had an mOS of 184 days (95% CI: 9-NE) compared with 100 days for those classified as having intermediate risk AML (95% CI: 57-NE), 98 days (95% CI: 79–153) for those classified as having adverse risk AML, and 21 days (95% CI: 4-NE) for patients whose 2022 ELN risk stratification could not be determined (*p* = 0.03; Table 1 and Figure 1c). Risk stratification as per the 2022 ELN classification could not be assessed in 12 patients. Patients classified as favorable risk by the 2024 ELN criteria unfit for intensive chemotherapy had an mOS of 112 days (95% CI: 95–184) compared with 147 days for those classified as intermediate risk (95% CI: 79–916), 50 days (95% CI: 36–162) for those classified as adverse risk, and 17 days (95% CI: 4-NE) for patients whose 2024 ELN risk classification could not be determined (*p* = 0.01; Table 1 and Figure 1d). Risk stratification as per the 2024 ELN classification could not be assessed in 13 patients. However, when adjusted for other covariates, there was no significant difference in mOS for the 2022 ELN or 2024 ELN risk stratification (Figure 2). AML-directed treatment was associated with a longer mOS across all 2022 ELN risk categories compared with no treatment following diagnosis, except among patients whose risk categorization could not be assessed (Table 2). Among patients who did not receive induction chemotherapy, AML-directed treatment was associated with a significantly longer mOS compared with no AML-directed treatment among those with favorable- and intermediate-risk disease according to the 2024 ELN risk stratification (Table 2). Among patients who were classified as having adverse risk AML based on the 2024 ELN risk stratification, mOS was 88 days with AML-directed treatment and 44 days without treatment (*p* = 0.2), with no significant association between AML-directed treatment and survival observed (Table 2). These associations were maintained when patients who did not receive treatment due to clinical deterioration were excluded from the analysis. No patients with adverse-risk disease based on the 2024 ELN risk classification were treated with induction chemotherapy.

Patients who were diagnosed with AML in the inpatient setting did not have a statistically significant difference in mOS compared with those diagnosed as outpatients (81 days, 95% CI: 44–120 vs. 126 days, 95% CI: 95–184; *p* = 0.6; Table 1). ASCT was associated with a longer mOS compared with no ASCT (253 days, 95% CI: 207-NE vs. 82 days, 95% CI: 65–110; *p* = 0.005; Table 1). Patients who were treated with induction chemotherapy had an mOS of 253 days (95% CI: 82-NE), compared with 100 days (95% CI: 64–184) for HMA monotherapy, 91 days (95% CI: 41-NE) for HMA and other targeted therapies, 158 days (95% CI: 98–282) for HMA and venetoclax, and 260 days with gemtuzumab ozogamicin (95% CI: NE-NE; *p* = 0.7; Table 1 and Figure 1e). After adjusting for other covariates, first-line treatment was not independently associated with overall survival (Figure 2). Patients who achieved a best response of CRc or a morphologic leukemia-free state (MLFS) had an mOS of 428 days (95% CI: 253–1180) compared with 98 days (95% CI: 81-NE) for those who had persistent disease (Table 1). Thirty-four patients who received treatment (44%) did not have a second bone marrow biopsy to assess for response, and this was largely due to clinical deterioration (68%).

Treatment with induction chemotherapy was associated with higher rates of CRc/MLFS (78%) compared with 47% with HMA/venetoclax (*p* = 1) and 22% with HMA monotherapy, although these differences were not statistically significant (*p* = 0.1). No patients treated with HMA alongside another targeted agent achieved CRc/MLFS.

### 3.2. Treatment Patterns

**Figure 3:**
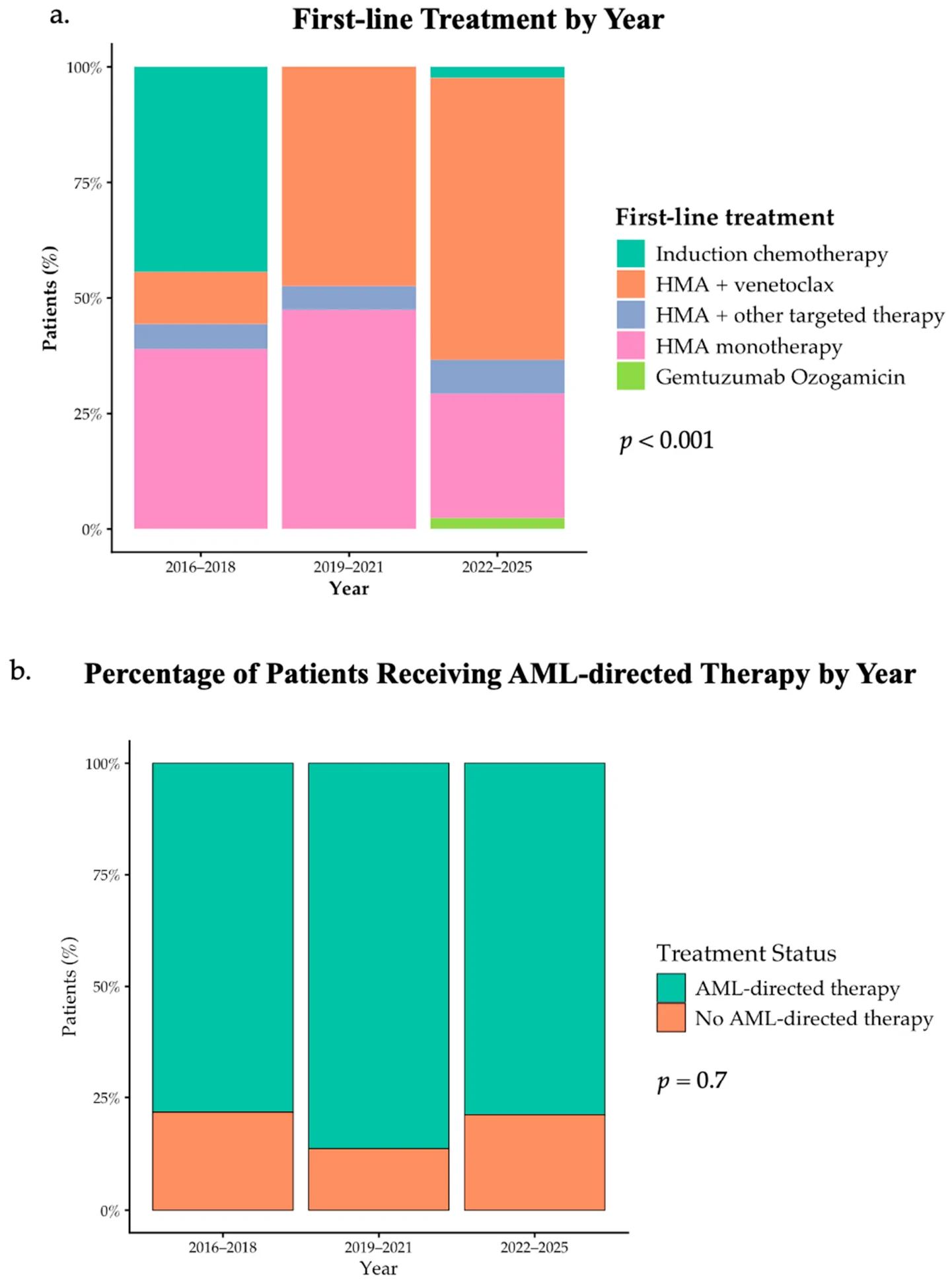
Percentage of patients receiving AML-directed therapy by year of diagnosis (**a**) and distribution of first-line treatment among treated patients by year of diagnosis (**b**). Abbreviations: AML = acute myeloid leukemia; HMA = hypomethylating agent.

The proportion of patients receiving AML-directed treatment over time has remained similar: 78% for those diagnosed in 2016–2018, 86% for 2019–2021, and 79% for 2022–2025 (*p* = 0.7; Figure 3a). In 2016–2018, 44% of patients who received AML-directed therapy were treated with induction chemotherapy. No patients were treated with induction chemotherapy in 2019–2021, and 3% were treated with induction chemotherapy in 2022–2025 (Figure 3b). In 2016–2018, 11% of patients received HMA/venetoclax as first-line treatment. By 2019–2021, 47% of patients were treated with HMA/venetoclax upfront, and in 2022–2025, 61% of patients were treated with HMA/venetoclax as the first-line treatment.

Nine patients were treated with induction chemotherapy, and one patient died during their induction cycle. Of the 36 patients treated with HMA/venetoclax, five patients discontinued therapy during the first cycle, including two patients who died during the first cycle. Three additional patients discontinued treatment during or after cycle 2, and 10 patients required dose reduction. Of the 27 patients treated with HMA monotherapy, six patients received one cycle of therapy or less, including five patients who died during the first cycle. Four patients underwent dose reduction, including one patient after CRi was achieved.

Seven patients underwent ASCT. Four of the seven patients were treated with HMA and venetoclax prior to transplant, and three patients were treated with standard induction chemotherapy. Patients who did not undergo ASCT were treated with a median of two cycles of therapy (IQR: 1–5). Patients with an ECOG PS of 0 or 1 received a median of three cycles of treatment (IQRs: 2–7 and 2–8, respectively) while patients with an ECOG PS of two received a median of one cycle of treatment (IQR: 1–5).

Of the 65 patients who received AML-directed therapy, but who did not undergo ASCT, 15 (23%) received subsequent treatment upon disease progression. Four patients remained on first-line treatment at the time of data cutoff. Two patients who were lost to follow-up were excluded. Of the patients with an ECOG PS of 0 at diagnosis, 43% of treated patients received subsequent therapy. For those with an ECOG PS of one at diagnosis, 15.6% of treated patients received subsequent therapy, and no patients with an ECOG PS of two or higher at diagnosis received second-line treatment. Eleven patients (28.2%) who were 75–79 years old at diagnosis received subsequent therapy compared with 10% of patients who were 80–85 years old at diagnosis and 5% of patients who were older than 85 years at diagnosis. These differences were not statistically significant.

### 3.3. Healthcare Utilization

Seventy-seven patients were included in the analysis of transfusion requirements, and 20 patients were excluded due to missing data. Treatment with HMA/venetoclax was associated with a higher median number of cumulative transfusions (30 transfusions, IQR: 9–60) following diagnosis compared with those who received induction chemotherapy (22 transfusions, IQR: 12–47; Table 3). However, treatment with HMA/venetoclax was associated with fewer transfusions during the first 30 days (median 7, IQR: 4–13) compared with those who received induction chemotherapy (median 21, IQR: 11–25; Table 3). These pairwise differences were not statistically significant, and this pattern persisted after excluding patients who underwent ASCT. In a sensitivity analysis accounting for differences in time at risk, there were no significant differences in median transfusion rates per month for patients at risk across first-line treatment groups (Supplemental Table S2). Mean transfusion data are provided in Supplemental Table S3.

Eighty-six patients were included in the analysis of inpatient days, and 11 patients were excluded due to missing data. In pairwise analyses, treatment with induction chemotherapy was associated with a higher median number of days in the hospital from diagnosis to death or censoring (85 days, IQR: 60–107) compared with treatment with HMA/venetoclax (38 days, IQR: 24–91, *p* = 0.09) and HMA monotherapy (28 days, IQR: 14–38, *p* = 0.003; Table 4). Twenty patients (23%) were admitted to the ICU, and these patients spent a median of 4 days in the ICU (IQR: 3–7). Median days spent in the ICU did not differ by first-line treatment (*p* = 0.8, Table 4), and the proportion of patients requiring an ICU level of care also did not differ by first-line treatment (*p* = 0.2). Mean inpatient and ICU days are provided in Supplemental Table S4. In a sensitivity analysis accounting for differences in time at risk, there were no significant differences in median inpatient days or ICU days per month at risk across the first-line treatment groups (Supplemental Table S2). Treatment with AML-directed therapy was associated with significantly more cumulative transfusions following diagnosis and significantly more days admitted to the hospital than patients who did not receive treatment. The median transfusion rate was higher among patients who received AML-directed therapy compared with those who did not (*p* = 0.05), while median inpatient and ICU days per month at risk did not differ between treated and untreated patients (Supplemental Table S2).

Of the 85 patients who were deceased at the data cutoff, 71 (84%) were enrolled in hospice care or were formally transitioned to CMO, including 37 (44%) who received inpatient or outpatient hospice care and 34 (40%) who were transitioned to CMO while hospitalized. Data were not available for one patient. The median number of days on hospice care or CMO was two (IQR: 1–5) for all patients (Table 4). Treated patients did not spend a significantly greater number of days receiving hospice care compared with untreated patients (2 days, IQR: 1–5 vs. 3 days, IQR: 1–22; *p* = 0.6, Table 4). There was also no significant difference in the median number of days receiving hospice care with first-line treatment. Mean palliative days are provided in Supplemental Table S4. Fifty-three percent of patients who were treated with AML-directed therapy and 42% of patients who did not receive any treatment following diagnosis died in the hospital (*p* = 0.44).

## 4. Discussion

Our single-center retrospective analysis of patients aged 75 years and older with AML suggests that survival remains poor in this older population. Although overall survival among patients with AML has improved over time with the expansion of therapeutic options and evolving management strategies, these improvements have not translated to the same extent among older adults. In older patients with AML, only a minority are sufficiently fit and eligible to undergo allogeneic stem cell transplantation, limiting the number of patients who may benefit from this approach and its potential for improved long-term outcomes [27].

While our study captured the shift in treatment patterns from intensive induction chemotherapy and HMA monotherapy to HMA and venetoclax over time, the proportion of patients able to receive treatment remained stable. Furthermore, older adults in our study received AML-directed therapy and transplant at much higher rates than reported in some large database studies [2,15,28,29]. This is consistent with prior findings that higher proportions of patients, including older patients, managed at academic centers received AML-directed treatment [2,30]. However, other differences between cohorts, including performance status, comorbidity burden, disease biology, and treatment selection, may also contribute to these differences. On a population level, older patients with AML are increasingly receiving AML-directed therapy in recent decades [2,15,29,31].

Our institution contributes data to the NCDB, and some overlap with our prior NCDB cohort is possible, although the extent of patient-level overlap cannot be determined. Our prior analysis of the NCDB was designed to characterize survival outcomes and treatment patterns among adults aged 80 years and older at the population level, whereas the current study provides more granular clinical data in adults aged 75 years and older. This includes first-line leukemia-directed treatment regimens and treatment patterns over time, ECOG PS, molecular and cytogenetic characteristics used for ELN risk stratification, transfusion utilization, hospital and ICU utilization, palliative care utilization, and place of death. These granular data provide complementary clinical detail beyond the population-level outcomes examined in our prior NCDB analysis.

Our results are overall comparable to what has been reported for patients 70 years and older using the SEER database [1]. Patients in our dataset had a slightly longer mOS despite being an older cohort, and this might be explained by a longer mOS seen in patients with AML treated at academic centers, including older patients [2,30].

Overall, among patients aged 75 years and older, AML-directed treatment was associated with longer mOS compared with no treatment following diagnosis, although this finding may be influenced by treatment selection bias. These findings suggest that older adults should not be excluded from AML-directed therapy when treatment is clinically appropriate. With increasing availability of lower-intensity approaches, careful individualized assessment of patients to identify those most likely to benefit from treatment is important. Our comparison of treated and untreated patients is also subject to potential immortal-time bias, as treatment status was defined as whether patients ever received AML-directed therapy, and survival was measured from the time of diagnosis. Thus, patients classified as treated necessarily survived until treatment initiation. Although a sensitivity analysis excluding patients who did not receive treatment due to early clinical deterioration yielded similar associations, this analysis does not eliminate potential immortal-time bias. These findings should therefore be interpreted as associations rather than causal estimates of treatment effect.

The shorter mOS observed among patients classified as favorable risk compared with those classified as intermediate risk according to the 2024 ELN criteria was unexpected and should be interpreted cautiously, particularly given the relatively small subgroup sizes and potential differences in first-line treatment. Within the ELN risk groups evaluated, AML-directed treatment was associated with longer mOS compared with no treatment, except among patients with 2024 ELN adverse-risk disease. These results are consistent with prior studies describing poor outcomes with venetoclax-based regimens in patients with *TP53*-mutated AML [5,6,10,32]. Although this pattern raises the possibility that the association between AML-directed therapy and survival may differ in 2024 ELN adverse-risk/*TP53*-mutated disease, this cannot be established from the present study because a formal treatment-by-ELN interaction was not assessed. The findings in the 2024 ELN adverse-risk/*TP53*-mutated subgroup should be interpreted cautiously given the small sample size and limited number of events, which may have reduced the power to detect a meaningful difference in survival. A clinically meaningful treatment benefit cannot be excluded, and larger studies are needed to better define treatment outcomes in this high-risk population. Older patients with 2024 ELN adverse-risk/*TP53*-mutated AML continue to experience poor outcomes with currently available therapies, and clinical trial enrollment may be particularly valuable for these patients when feasible. When investigational therapies are not available or appropriate for patients who are not eligible for ASCT, best supportive care may represent an appropriate treatment approach for some older patients with ELN 2024 adverse-risk/*TP53*-mutated AML, depending on individual clinical circumstances and treatment goals.

ECOG PS may warrant further investigation as a predictor of shorter mOS and fewer treatment cycles. Unlike in other hematologic malignancies, poor PS in older adults with AML may not be readily reversible; however, further studies are needed to validate this finding given the limited sample size of the present study [33,34,35]. Missing ECOG performance status may have introduced selection bias or residual confounding, given the potential influence of performance status on both treatment decisions and survival.

While VIALE-A did not report mOS for the subgroup of patients aged 75 years and older, mOS for the entire trial population including older patients and those unfit for intensive chemotherapy was 9.6 months in the azacitidine monotherapy arm and 14.7 months in the azacitidine and venetoclax arm [7]. A pooled analysis for VIALE-A and the phase Ib M14–358 studies showed an mOS of 8–10 months for patients 75 years and older treated with azacitidine monotherapy and 12–14 months for older patients treated with azacitidine and venetoclax [36]. In the VIALE-A subgroup analysis, patients aged 75 years or older treated with azacitidine plus venetoclax had a 46% lower risk of death than those treated with azacitidine alone (HR 0.54, 95% CI: 0.39–0.73) [7]. The pooled analysis by Venditti et al. showed a longer mOS in patients aged 75 years and older treated with azacitidine and venetoclax compared with azacitidine monotherapy [36].

While some real-world cohort studies have validated the results of VIALE-A in older adults, the data have been generally mixed [22,23,24]. One meta-analysis of 47 studies in adults 55 years and older with newly diagnosed AML ineligible for intensive chemotherapy found that addition of venetoclax to HMA probably reduces mortality with moderate certainty evidence [25]. Another meta-analysis of 24 studies evaluating HMA with and without venetoclax showed that while response rates were similar to those reported in the VIALE-A, mOS was considerably worse in real-world cohorts, although this study did not focus specifically on older adults [24]. This meta-analysis also found that in real-world studies, treatment with HMA/venetoclax achieved a significantly higher CRc rate; however, there was no significant survival benefit to using HMA/venetoclax versus HMA monotherapy [24]. Our findings were directionally consistent with these results, as HMA/venetoclax was not associated with longer mOS compared with HMA monotherapy in our cohort. Treatment selection bias may have influenced these findings, as treatment assignment was not randomized and may have been influenced by factors such as patient fitness, comorbidities, and disease biology. Preferential administration of venetoclax to fitter patients would be expected to bias the analysis toward an observed survival advantage with HMA/venetoclax. Despite this potential bias, HMA/venetoclax was not associated with longer mOS compared with HMA monotherapy in our cohort. However, the small sample size may have limited our ability to detect a survival difference, and residual unmeasured confounding cannot be excluded.

There may be several explanations for the difference in findings between our real-world data and VIALE-A. Patients enrolling on clinical trials are likely to be significantly more fit with better support and fewer barriers to care, although this is difficult to capture in a retrospective analysis. Patients in our study who were treated with HMA monotherapy received azacitidine, decitabine, and decitabine/cedazuridine, with most patients receiving decitabine (59%). Meta-analysis data suggest that decitabine and venetoclax are superior to decitabine alone, although the quality of evidence was low [24]. This is further confounded by variations in decitabine dosing in clinical practice. mOS was numerically higher in the HMA/venetoclax group, and the small sample size may have lacked statistical power to demonstrate a statistically significant benefit.

Patients treated with HMA and venetoclax in our study cohort received more cumulative transfusions, although fewer transfusions within the first 30 days and first 60 days following diagnosis, compared with patients treated with induction chemotherapy. While the median number of cycles of HMA and venetoclax received by patients in this study was low, a small number of patients were treated with a higher number of cycles (up to 15) and required high numbers of total transfusions. Differences in cumulative healthcare utilization, including transfusion burden, should be interpreted in the context of differential survival, as patients with longer survival have greater opportunity to accumulate utilization events over time. Although transfusion requirements were assessed at 30 and 60 days following diagnosis, we did not perform analogous time-limited analyses for inpatient days or ICU days. Patients with an ECOG PS of 1 or higher at diagnosis were unlikely to receive any subsequent lines of therapy, underscoring the narrow window during which patients remain candidates for additional therapy.

We found no difference in days on hospice or CMO for treated and untreated patients, suggesting that delaying hospice enrollment in favor of a trial of palliative therapy did not decrease the duration of hospice time. Low utilization of hospice in AML and other hematologic malignancies has been well documented, with transfusion dependence as a primary barrier to hospice enrollment [37,38,39,40,41,42,43]. The proportion of patients who were on hospice or receiving CMO at end of life was higher in our study population compared with what has been reported in other studies. However, over 50% of patients died in the hospital, and the median number of palliative days remained low for patients who received AML-directed treatment and those who did not. This likely reflects the use of inpatient hospice services and inpatient CMO care at the end of life. Additional factors that may have influenced hospice utilization and duration include institutional practice patterns, access to outpatient hospice resources, and socioeconomic factors. These factors were not systematically captured in our retrospective study. These findings should be interpreted in the context of our single-institution experience and may not be generalizable to other healthcare settings. Although these findings may not be generalizable beyond our institution, the observed healthcare utilization patterns are broadly consistent with those reported in other institutional studies [44]. These findings raise the hypothesis that substantial healthcare utilization and intensive end-of-life care remain common among older adults with AML despite the availability of novel therapies, although our study cannot determine whether newer treatments have changed healthcare utilization over time.

This study had several limitations. Due to its retrospective, single-center design, there was potential for selection bias and information bias, although, when possible, data subject to obvious information bias were excluded from analysis. The single-institution design also limited generalizability of these data to other practice settings and patient populations. The relatively small sample size limited statistical power, particularly for subgroup analyses. Treatment and response findings should therefore be interpreted in the context of the limitations inherent to retrospective observational data, as described above.

Although the multivariable analysis adjusted for first-line treatment, the 2024 ELN risk group, ECOG PS, age at diagnosis, year of diagnosis, and inpatient versus outpatient diagnosis, treatment assignment in this retrospective cohort was not randomized and was influenced by clinical decision-making. Other factors, including comorbidities and measures of frailty not captured by ECOG PS, may also have influenced treatment selection and survival. Thus, residual confounding and selection bias cannot be excluded, and the observed associations between treatment and survival should not be interpreted as causal treatment effects.

Response assessment was limited, as nearly half of treated patients did not undergo a second bone marrow biopsy, precluding formal assessment of treatment response. However, 68% of patients who did not undergo repeat bone marrow evaluation experienced significant clinical decline. Although these patients could not be assigned a formal response category using 2022 ELN criteria, the absence of repeat marrow evaluation in this setting provides clinically relevant information regarding their treatment course. Therefore, the reported response rates should be interpreted with caution and in the context of the substantial proportion of patients who were not formally evaluable for response.

Despite the relatively small sample size, a detailed review of the medical record allowed us to characterize reasons AML-directed therapy was not administered, including both patient decisions and provider recommendations against treatment because of medical comorbidities or clinical instability, which has been rarely described.

## 5. Conclusions

This study highlights the particular vulnerability of older patients with AML and underscores the ongoing need for novel therapeutic approaches that offer meaningful clinical benefit while maintaining acceptable tolerability. Treatment of AML in older adults remains a complex clinical challenge, as comorbidities and functional limitations can restrict treatment options and tolerability. Older adults with AML continue to experience poor survival and substantial healthcare utilization near the end of life, emphasizing the importance of both effective treatment strategies and supportive care. With the increasing availability of less toxic induction regimens, early identification of patients who may benefit from these strategies is critical. Older adults should be better represented in AML clinical trials, and eligibility criteria should be reconsidered to permit enrollment of populations that more closely reflect those affected by AML in routine clinical practice, as outcomes observed in clinical trials may not fully reflect real-world outcomes.

Beyond the development of novel therapeutics, improvements in supportive and end-of-life care represent an important opportunity to enhance quality of life for older patients with AML. Expanding access to transfusions within hospice settings may allow more patients with AML to receive comfort-focused care without sacrificing symptom control or delaying hospice enrollment. This approach may also decrease hospital admissions and high-cost interventions near the end of life. Future research should evaluate models of hospice care that incorporate transfusion support to better define their impact on quality of life, symptom burden, and healthcare utilization.

## Figures and Tables

**Table 1:** Median overall survival by baseline demographics and clinical characteristics.

		N	mOS (Days)	95% CI (Days)	*p*-Value
All patients		97	95	78–147	NA
Sex	Male	60	98	65–153	0.2
	Female	37	95	80–264	
Age	75–79y	47	98	65–206	0.5
	80–84y	31	100	50–184	
	85y+	19	95	44–260	
Treatment status	AML-directed therapy	78	144	98–184	<0.001
	No AML-directed therapy	19	17	7–50	
Year of diagnosis	2016–2018	23	82	64–428	0.9
	2019–2021	22	104	69–166	
	2022–2025	52	98	44–184	
ECOG performance status at diagnosis	0	9	207	158–NE	0.02
	1	36	120	95–253	
	2	16	78	41–NE	
	3	1	25	NE–NE	
	4	3	81	17–NE	
	Unavailable	32	27	11–82	
2022 ELN	Favorable	9	184	9–NE	0.03
	Intermediate	14	100	57–NE	
	Adverse	62	98	79–153	
	Unable to assess	12	21	4–NE	
2024 ELN ^1^	Favorable	34	112	95–184	0.01
	Intermediate	21	147	79–916	
	Adverse	20	50	36–162	
	Unable to assess	13	17	4–NE	
Inpatient diagnosis	Yes	58	81	44–120	0.6
	No	39	126	95–184	
First line treatment	Induction chemotherapy	9	253	82–NE	0.7
	HMA	27	100	64–184	
	HMA and other targeted therapy	5	91	41–NE	
	HMA and venetoclax	36	158	98–282	
	Gemtuzumab ozogamicin	1	260	NE–NE	
Transplant status	ASCT	7	253	207–NE	0.005
	No ASCT	88	82	65–110	
Best Response	CRc/MLFS	31	428	253–1180	<0.001
	Persistent Disease	13	98	81–NE	
	Not assessed	34	65	29–104	
	N/A (no AML-directed therapy)	19	17	7–This h50	

Abbreviations: AML = acute myeloid leukemia; mOS = median overall survival; CI = confidence interval; ELN = European LeukemiaNet; ECOG = Eastern Cooperative Oncology Group; HMA = hypomethylating agent; ASCT = allogeneic stem cell transplant; CRc = composite complete response; MLFS = morphologic leukemia-free status; NE = not estimable; NA = not applicable. ^1^ Patients who received intensive chemotherapy were excluded.

**Table 2:** Median overall survival by ELN risk stratification and treatment status.

Risk Stratification	Risk Category	AML-Directed Therapy	N	mOS (Days)	95% CI (Days)	*p*-Value
ELN 2022	Favorable	Yes	6	340.5	184–NE	0.006
		No	3	5	2–NE	
	Intermediate	Yes	13	184	57–NE	0.04
		No	1	7	NE–NE	
	Adverse	Yes	54	114	95–164	<0.001
		No	8	40	27–NE	
	Unable to assess	Yes	5	78	4–NE	0.2
		No	7	17	3–NE	
ELN 2024 ^1^	Favorable	Yes	30	144	104–341	<0.001
		No	6	9	3–NE	
	Intermediate	Yes	25	147	81–NE	0.009
		No	1	7	NE–NE	
	Adverse	Yes	15	88	34–NE	0.2
		No	5	44	36–NE	
	Unable to assess	Yes	5	153	15–NE	0.1
		No	8	11	3–NE	

Abbreviations: AML = acute myeloid leukemia; mOS = median overall survival; CI = confidence interval; ELN = European LeukemiaNet; NE = Not estimable. ^1^ Patients who received intensive chemotherapy were excluded.

**Table 3:** Median transfusion requirements following diagnosis by treatment status and first-line treatment.

		N	Total Number of Transfusions	*p*-Value	Transfusions First 30 Days	*p*-Value	Transfusions First 60 Days	*p*-Value
All patients		77	10 (2–9)	NA	4 (1–8)	NA	6 (2–16)	NA
Treatment Status	AML-directed therapy	58	24 (6–47)	<0.001	6 (3–13)	<0.001	10 (5–20)	<0.001
	No AML- directed therapy	19	1 (0.5–2)		1 (0–2)		2 (1–2)	
First-line treatment	Induction chemotherapy	3	22 (12–47)	<0.001	21 (11–25)	<0.001	22 (12–43)	<0.001
	HMA	19	23 (4–39)		5 (2–9)		5 (3–20)	
	HMA and other targeted therapy	5	10 (8–13)		1 (1–8)		11 (7–19)	
	HMA and venetoclax	26	30 (9–60)		7 (4–13)		14 (7–21)	
	Gemtuzumab ozogamicin	1	24 (24–24)		4 (4–4)		6 (6–6)	
	No AML-directed therapy	19	1 (1–2)		1 (0–2)		1 (1–2)	

Interquartile range reported in parentheses. Abbreviations: AML = acute myeloid leukemia; HMA = hypomethylating agents; NA = not applicable.

**Table 4:** Median healthcare utilization (inpatient, ICU, and palliative care days) by treatment status, transplantation, and first-line treatment.

		Total Days Admitted	*p*-Value	Total ICU Days ^1^	*p*-Value	Total Palliative Days	*p*-Value
All patients		26(13–54)	NA	4 (3–7)	NA	2 (1–5)	NA
Treatment Status	AML-directed therapy	36 (19–75)	<0.001	4 (3–7)	0.7	2 (1–5)	0.6
	No AML-directed therapy	11 (7–16)		3 (3–3)		3 (1–22)	
Transplant Status	ASCT	98 (88–116)	<0.001	3 (3–4)	0.2	1 (1–1)	0.08
	No ASCT	24 (12–46)		4 (3–7)		3 (1–6)	
First-line treatment	Induction chemotherapy	85 (60–107)	<0.001	4 (4–4)	0.8	1 (0–3)	0.5
	HMA	28 (14–38)		7 (3–7)		2 (1–7)	
	HMA and other targeted therapy	24 (11–54)		7 (5–9)		16 (7–24)	
	HMA and venetoclax	38 (24–91)		3 (2–5.5)		2 (1–4)	
	Gemtuzumab ozogamicin	32 (32–32)		NA		2 (2–2)	
	No AML-directed therapy	11 (7–16)		3 (3–3)		3 (1–22)	

Interquartile range reported in parentheses. Abbreviations: AML = acute myeloid leukemia; HMA = hypomethylating agents. ^1^ Patients who did not require any ICU admissions were excluded.

## Data Availability

The database used in this analysis was developed by the authors and can be available upon request.

## Supplementary Materials

The following supporting information can be downloaded at: https://www.mdpi.com/article/10.3390/cancers18193230/s1, Table S1: Median event free survival by baseline demographics and clinical characteristics; Table S2: Healthcare utilization rates accounting for time at risk; Table S3: Mean transfusion requirements following diagnosis by treatment status and first-line treatment; Table S4: Mean healthcare utilization (inpatient, intensive care, and palliative care days) by treatment status, transplantation, and first-line treatment.
