# Supplementary material for: Contemporary Clinical Outcomes and Healthcare Utilization in Older Adults with Acute Myeloid Leukemia: Insights from a Single-Center Retrospective Cohort
Source: Cancers (Basel). 2026 Oct 7;18(19):3230. doi: 10.3390/cancers18193230 (PMC13664294; doi:10.3390/cancers18193230)
Supplement: Supplementary file 1 [file cancers-18-03230-s001.zip › cancers-4561877-supplementary.pdf]

Supplemental Materials

Table S1. Median event free survival by baseline demographics and clinical characteristics

		N	EFS (days)	95% CI (days)	p-value
All patients		97	87	65–142	NA
Sex	Male	60	79	50–147	0.2
	Female	37	95	57–264	
Age	75-79y	47	82	57–158	0.4
	80-84y	31	100	48–166	
	85y+	19	88	44–162	
Treated	Treated	78	140	88–162	<0.001
	Untreated	19	17	7–50	
Year of diagnosis	2016-2018	23	69	33–428	0.9
	2019-2021	22	94.5	65–166	
	2022-2025	52	88	40–157	
ECOG at diagnosis	0	9	207	158–NE	0.002
	1	36	120	95–253	
	2	16	78	41–NE	
	3	1	25	NE–NE	
	4	3	81	17–NE	
	NA	32	27	11–72	
ELN 2022	Favorable	9	184	9–NE	0.035
	Intermediate	14	100	57–NE	
	Adverse	62	98	79–153	
	Unable to assess	12	21	4–NE	
ELN 2024	Favorable	29	95	58–151	0.05
	Intermediate	18	140	37–NE	
	Adverse	18	44	34–120	
	Unable to assess	12	21	4–NE	
Inpatient diagnosis	Yes	58	58	34–120	0.4
	No	39	127	88–161	
First line treatment	Induction	9	161	78–NE	0.8
	HMA	27	88	55–157	

	HMA and other targeted agent	5	69	37–NE	
	HMA and venetoclax	36	144	94–264	
	Gemtuzumab ozogamicin	1	260	NE–NE	
Transplant Status	ASCT	7	2682	207–NE	0.002
	No ASCT	88	78	50–100	
Best Response	CR/MLFS	31	373	253–657	<0.001
	Persistent Disease	13	58	37–NE	
	Not assessed	34	65	29–100	
	NA (no treatment)	19	17	7–50	

Abbreviations: AML = acute myeloid leukemia; EFS = event free survival; CI = confidence interval; ELN = European LeukemiaNet; ECOG = Eastern Cooperative Oncology Group; HMA = hypomethylating agent; ASCT = allogeneic stem cell transplant; CRc = composite complete response; MLFS = morphologic leukemia free status, NE = not estimable

[1]Patients who received intensive chemotherapy were excluded

Table S2. Healthcare utilization rates accounting for time at risk

		**Median transfusions per month at risk**	**p-value**	**Median inpatient days/month at risk**	**p-value**	**Median ICU days per month at risk**	**p-value**
Treatment status	AML-directed therapy	N = 58; 5.2 (2.0–11.3)	0.06	N = 67; 8.2 (3.7–19.7)	0.05	N = 67; 0 (0–0.1)	0.05
	No AML-directed therapy	N = 19; 1.8 (0.2–11.2)		N = 19; 20.3 (5.4–67.0)		N = 19; 0 (0–0)	
First line treatment	Induction	N = 3; 7.6 (4.4–17.9)	0.7	N = 9; 11.8 (3.9–27.7)	0.8	N = 9; 0 (0–0)	0.5
	HMA	N = 19; 4.5 (1.8–9.7)		N = 20; 8.3 (1.7–23.8)		N = 20; 0 (0–0.1)	
	HMA and other targeted agent	N = 5; 4.9 (3.0–5.9)		N = 5; 15.4 (8.2–20.3)		N = 5; 0 (0–0.6)	
	HMA and venetoclax	N = 30; 7.2 (1.8–9.7)		N = 32; 6.9 (4.0–18.3)		N = 32; 0 (0–0.2)	

Interquartile range reported in parentheses. N varies by utilization measure due to missing data.

Abbreviations: ICU = intensive care unit, HMA = hypomethylating agent

Table S3. Mean transfusion requirements following diagnosis by treatment status and first-line treatment

		N	Total number of transfusions (mean)	Transfusions first 30 days (mean)	Transfusions first 60 days (mean)
All patients		77	26.4 (35.4)	7.1 (8.2)	11.2 (12.9)
Treatment status	AML-directed therapy	58	34.5 (37.5)	8.9 (8.7)	14.4 (13.5)
	No AML-directed therapy	19	1.7 (1.9)	1.7 (1.9)	1.7 (1.9)
First line treatment	Induction chemotherapy	3	31.7(1.9)	16.7 (14.0)	28.7 (31.5)
	HMA	19	24.4 (26.7)	6.7 (6.3)	10.0 (9.5)
	HMA and other targeted agent	5	17.0 (20.6)	6.6 (9.1)	15.0 (13.8)
	HMA and venetoclax	26	40.5 (41.6)	10.5 (9.6)	16.8 (13.6)
	Gemtuzumab ozogamicin	1	24.0 (NA)	4.0 (NA)	6.0 (NA)

	No AML-directed therapy	19	1.7 (1.9)	1.7 (1.9)	1.7 (1.9)

Standard deviation reported in parentheses

Abbreviations: AML = acute myeloid leukemia; HMA = hypomethylating agents

Table S4. Mean healthcare utilization (inpatient, intensive care, and palliative care days) by treatment status, transplantation, and first-line treatment

		**Total days admitted**	**Total ICU days**	**Total palliative days**
All patients		40.8	5 (3.2)	7.3 (14)
Treatment status	AML-directed therapy	48.9 (40.4)	5.1 (3.2)	5.6 (11.5)
	No AML-directed therapy	12.2 (10.3)	3.0 (NA)	13.0 (19.7)
Transplant status	ASCT	34.4 (31.4)	3.0 (1.4)	0.67 (0.6)
	No ASCT	107 (57.8)	5.4 (3.3)	7.73 (14.3)
First line treatment	Induction	92.8 (21.6)	4.0 (NA)	2.9 (4.7)
	HMA	29.4 (21.6)	5.6 (3.0)	7.9 (16.9)
	HMA and other targeted agent	36.6 (31.9)	7.0 (5.7)	15.5 (12.8)
	HMA and venetoclax	51.2 (36.4)	4.5 (3.3)	3.4 (5.5)

	Gemtuzumab ozogamicin	32 (NA)	NA	2.0 (NA)
	NA	12.2 (10.3)	3.0 (NA)	13.0 (19.7)

Standard deviation reported in parentheses

Abbreviations: AML = acute myeloid leukemia; ICU = intensive care unit; HMA = hypomethylating agents; NA = not applicable

[1]Patients who did not require any ICU admissions were excluded
